# Individual Behaviors and COVID-19 Lockdown Exit Strategy: A Mid-Term Multidimensional Bio-economic Modeling Approach

**DOI:** 10.3389/fpubh.2020.606371

**Published:** 2020-11-17

**Authors:** Ahmed Ferchiou, Remy Bornet, Guillaume Lhermie, Didier Raboisson

**Affiliations:** Université de Toulouse, INRA, ENVT, Toulouse, France

**Keywords:** bioeconomic model, public health, SIR, COVID-19, policy simulation

## Abstract

As of mid-2020, eradicating COVID-19 seems not to be an option, at least in the short term. The challenge for policy makers consists of implementing a suitable approach to contain the outbreak and limit extra deaths without exhausting healthcare forces while mitigating the impact on the country's economy and on individuals' well-being. To better describe the trade-off between the economic, societal and public health dimensions, we developed an integrated bioeconomic optimization approach. We built a discrete age-structured model considering three main populations (youth, adults and seniors) and 8 socio-professional characteristics for the adults. Fifteen lockdown exit strategies were simulated for several options: abrupt or progressive (4 or 8 weeks) lockdown lift followed by total definitive transitory final unlocking. Three values of transmission rate (Tr) were considered to represent individuals' barrier gesture compliance. Optimization under constraint to find the best combination of scenarios and options was performed on the minimal total cost for production losses due to contracted activities and hospitalization in the short and mid-term, with 3 criteria: mortality, person-days locked and hospital saturation. The results clearly show little difference between the scenarios based on the economic impact or the 3 criteria. This means that policy makers should focus on individuals' behaviors (represented by the Tr value) more than on trying to optimize the lockdown strategy (defining who is unlocked and who is locked). For a given Tr, the choices of scenarios permit the management of the hospital saturation level with regard to both its intensity and its duration, which remains a key point for public health. The results highlight the need for behavioral or experimental economics to address COVID-19 issues through a better understanding of individual behavior motivations and the identification of ways to improve biosecurity compliance.

## Introduction

Coronavirus disease 2019 (COVID-19) represents a change in paradigm for our society and the health care system. In recent decades, outbreaks have been maintained locally and have been limited over time, which makes COVID-19 a novel entity (1). As of mid-2020, eradicating a disease such as COVID-19 seems not to be an option, at least in the short term. The challenge for policy makers consists of implementing a suitable approach that contains the outbreak, limits extra deaths, and avoids the exhaustion of healthcare forces while mitigating the impact on the country's economy and on individuals' well-being (2). This means considering several competing objectives at the same time and continuously adapting the strategy and rules. The situation represents an economic dynamic optimization problem under constraint in an uncertain environment. Bioeconomic sequential optimization may help to find the best middle-term solutions that integrate the compromises between competing criteria. Epidemiologic and bioeconomic modeling provide a scientific background for evidence-based policy to be implemented in the societal, economic, and public health dimensions.

The constraints linked to COVID-19 arise both from the characteristics of the outbreak (epidemiologic parameters, severity of infection) and from the structure of the healthcare system (number of available hospital beds, testing facilities, personnel) (3–7). Economic constraints overtake biological constraints as the crisis extends, especially when the disease becomes endemic. Business resumption and diminished social welfare call into question both the cost-effectiveness of the policy and its acceptability for individuals in a compromise between the resumption of activities and public health (8–10). In addition to these standard constraints for decision-making, policymakers must address the biological uncertainty of a new virus (i.e., treatment, vaccine availability, immunity duration, relapse) and economic uncertainty (lockdown impact with large-scale shock and resilience of the social-ecological system). However, neither citizens nor policymakers like to deal with uncertainty. This situation justifies lean management that is adjustable in the short, middle and long term.

The lockdown and lockdown lift strategies differ by country, regardless of the country's sociodemographic characteristics. Up to mid-2020, the short- and middle-term strategies adopted prioritized public health outcomes while considering economic and societal (well-being) constraints. The middle- and long-term strategies will likely differ from the short-term strategies for countries that initially highlighted safety-first (strict lockdown), which may limit the economic and psychological consequences of the previous strategies, or for other countries with very light initial lockdown, which may now increase population protection and face high political risk.

A recent review highlighted the 5 key factors that have led to contractions in activity (and economy): direct losses due to death and infections, losses due to government policies such as lockdown and restrictions, declines in household consumption, local interactions within supply chains and trade, and possible hysteria effects that prevent a return to pre-crisis economic equilibrium (11). Gollier (12) noted that a limitation of many studies focused on COVID-19 lies in the way uncertainty is accounted for and the decrease in the studies' relevance with time. Macroeconomic studies focusing on international or national issues tend to assess past and/or future impacts of pandemics (13–16). They may or may not include solutions and suggestions to mitigate future impacts (17). Other economic studies focus on firms' strategies to limit the crisis impact; to date, these studies have underestimated impacts such as mental health (18). Observational or simulation-based studies based on sociology, psychology and economic approaches emphasize the efficiency of measures to change individuals' behavior and modulate outbreak dynamics (19–21).

Some bioeconomic studies deal with the trade-off between alternatives to control COVID-19, such as waiting for a vaccine, developing herd immunity, contact restrictions and, more broadly, all non-pharmaceutical interventions (22, 23). Interestingly, few bioeconomic optimization approaches that combine epidemiologic and economic approaches and accounting for multi-criteria decisions are available for COVID-19. Optimizing lockdown policies in India has been proposed using reinforcement learning (24).

A targeted lockdown lift strategy may help to achieve multiple objectives simultaneously and to find the trade-off between societal, economic, and public health criteria (2). We propose an empirical application of such an integrated bioeconomic optimization approach. With the example of the fourth largest French city, we model the lockdown lift under different scenarios and evaluate the best long-term strategies to highlight which political levers should preferentially focus on minimizing long-term impact.

## Materials and Methods

A bioeconomic model was developed to support the long-term lockdown lift strategy for Toulouse, a French city with 475,000 inhabitants. The model consists of an epidemiologic compartmental model that mimics epidemic dynamics and an economic optimization model that accounts for both monetary impact (local gross domestic product (GDP) and medical care costs) and medical staff and citizen welfare. The bio-economic approach considers both demographic and socio-professional profiles of the inhabitants and is focused on the trade-offs between economic impact limitations and the welfare of different groups of citizens.

### Epidemiologic Compartmental Model

**Figure 1:**
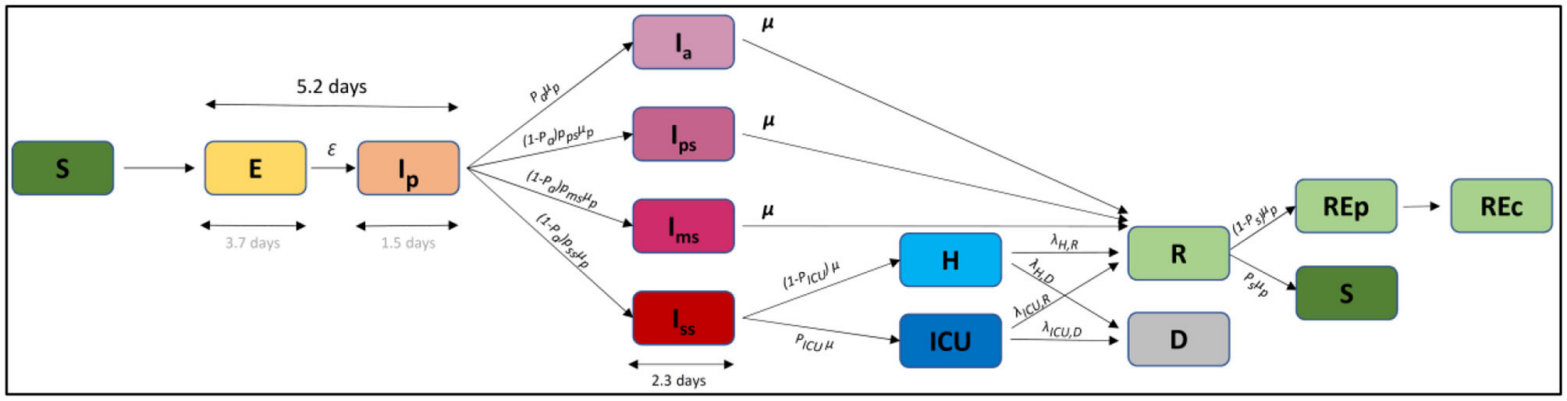
Compartmental model. S, susceptible; E, exposed; Ip, infectious in the prodromic phase; Ia, asymptomatic infectious; Ips, paucisymptomatic infectious; Ims, symptomatic infectious with mild symptoms; Iss, symptomatic infectious with severe symptoms; ICU, severe case admitted to ICU; H, severe case admitted to the hospital but not in intensive care; Rep, recovered without economic activity; Rep, recovered with economic activity; D, deceased.

We built a deterministic discrete age-structured model, considering the demographic and age profile share of the population (younger than 18 years old, adults, and seniors) based on the work performed by Di Domenico et al. (25). The compartmental model is described in Figure 1. In brief, individuals are divided into susceptible, exposed, infectious, hospitalized, in intensive care units (ICUs), recovered, and deceased. A prodromic phase is considered before the appearance of symptoms. During this phase, individuals have a smaller transmission rate (Tr) with respect to symptomatic individuals. During the second step of the infectious phase, individuals may remain asymptomatic (*I*_*a*_) or develop different degrees of severity of symptoms. Individuals may remain paucisymptomatic (*I*_*ps*_) or face mild (*I*_*ms*_) or severe (*I*_*ss*_) symptoms. Asymptomatic individuals (including children) have a smaller transmission rate (Tr) than symptomatic individuals. Children are assumed to become either asymptomatic or paucisymptomatic only and are considered to be as susceptible as adults. The recovery stage has been divided into recovery from an epidemiologic point of view (REp), meaning staying at home after the disease, and from an economic point of view (REc), meaning returning to work (with the same current rules at this time). After infection, a small part of the population (*P*_*s*_ = 10%) is considered to be susceptible again.

Three main populations were considered for the epidemiologic approach (young, adults, and seniors), and the model was refined by adding the socio-professional characteristics of the adults to account for differential lockdown exit strategies on this subpopulation (Table 1). The categories “medical” and “essential” workers were created, representing 30% of the whole active population. Students and unemployed subpopulations were also created since their movement and contacts were expected to differ from other adult populations during the lockdown and lockdown lift (26, 27). Four other socio-professional categories were created (26) based on (i) the impossibility of having at least partial remote work (denoted *Fixed*) and a simplification of the official socio-professional classification: lower supervisory and technical occupations (denoted Lower), intermediate occupations (denoted *Intermediate*), and higher managerial, administrative, and professional occupations (denoted *Higher*). Small employers and individual entrepreneurs were not a specific category since they fall into either the fixed or the intermediary category. Similarly, lower managerial, administrative and professional occupations were not distinguished from other lower occupation profiles and were included in the Lower category.

**Table 1:** Description of the socio-professional categories and the lockdown exit scenarios.

		**Young (<18 year)**	**Students**	**Unemployed**	**Seniors**	**Medical**	**Essentials**	**Active_** **lower**	**Active_** **fixed**	**Active_** **Intermediate**	**Active_higher**
Class of epidemiologic risk category		Child	Adult	Adult	Seniors	Adult	Adult	Adult	Adult	Adult	Adult
Inhabitants number		80,500	75,000	43,500	64,500	26,775	26,775	33,500	41,650	41,650	41,650
ActRelease_Popi, Lj_	L0	3%	3%	3%	3%	100%	100%	3%	3%	3%	3%
	L1	3%	3%	3%	3%	100%	100%	50%	50%	50%	50%
	L2	3%	3%	3%	3%	100%	100%	75%	75%	75%	75%
	L3	3%	3%	3%	3%	100%	100%	100%	50%	50%	50%
	L4	3%	3%	3%	3%	100%	100%	50%	100%	100%	50%
	L5	3%	3%	3%	3%	100%	100%	50%	50%	50%	100%
	L6	100%	3%	25%	20%	100%	100%	50%	50%	50%	50%
	L7	100%	3%	25%	20%	100%	100%	75%	75%	75%	75%
	L8	100%	3%	25%	20%	100%	100%	100%	50%	50%	50%
	L9	100%	3%	25%	20%	100%	100%	50%	100%	100%	50%
	L10	100%	3%	25%	20%	100%	100%	50%	50%	50%	100%
	L11	100%	3%	25%wc	25%wc	100%	100%	40%wc	40%wc	40%wc	40%wc
	L12	100%	3%	25%wc	25%wc	100%	100%	75%hwc	75%hwc	75%hwc	75%hwc
	L13	100%	3%	25%wc	25%wc	100%	100%	75%hwc	40%wc	40%wc	40%wc
	L14	100%	3%	25%wc	25%wc	100%	100%	40%wc	75%hwc	75%hwc	40%wc
	L15	100%	3%	25%wc	25%wc	100%	100%	40%wc	40%wc	40%wc	75%hwc
	L99	100%	100%	100%	100%	100%	100%	100%	100%	100%	100%

*ActRelease_Popi, Lj_ is the percentage of activity released for scenario L j and population i. For L11 to L16, wc, and hwc represent containment of contacts within categories (wc) or half containment of contacts within categories (hwc)*.

The biological model is based on the principle that contacts within and between the subpopulations are modulated during the lockdown and thereafter depend on the lockdown lift scenarios. The likelihood of becoming *Exposed* (Figure 1) consequently depends on the contact matrix and the transmission rate (i.e., probability of becoming *Exposed* if in contact with an *Infectious* person). The number of simulated contacts during lockdown and for each lockdown scenario were defined in relation to the percentage or activity released, as indicated in Equation (1):

(1) $$\begin{array}{l} \text{Contact}{\text{s}}_{\text{Pop}{\text{i}}^{*}\text{Pop}{\text{i}}^{\text{′}},\text{Lj}}=\text{CoefContact }*\text{ Contac}{\text{t}}_{\text{Pop}{\text{i}}^{*}\text{Pop}{\text{i}}^{\text{′}},\text{Init}}\\ \;*\text{ ActReleas}{\text{e}}_{\text{Popi},\text{Lj}} \end{array}$$

where

${\text{Contacts}}_{\text{Pop}{\text{i}}^{*}\text{Pop}{\text{i}}^{\text{′}},\text{Lj:}}$ contact matrix for the scenario L *j* and the populations *i* and *i'*
CoefContact: ponderation of the initial contact matrix due to change in behavior with time
${\text{Contact}}_{\text{Pop}{\text{i}}^{*}\text{Pop}{\text{i}}^{\text{′}},\text{Init:}}$ initial contact matrix for the populations *i* and *i'*
ActRelease_Popi, Lj_: percentage of activity released for scenario L*j* and population *i*.

### Lockdown and Lockdown Lift Scenarios

In the lockdown scenario (L0, Table 1), all subpopulations are locked (3% of released activity in terms of contacts) except medical and essential workers. This represents the policy implemented in France in phase 1, from March 18th to May 11th, 2020 (28). Schools were closed, and 70% of non-essential workers worked remotely.

In phase 2, starting May 11th, three sets of monitored lockdown lift strategies (L1-L5, L5-L10, and L11-15) were simulated (Table 1) and applied. For all scenarios, medical, and essential workers remained unlocked. In scenarios L1 to L5, all non-active subpopulations remained locked, and the 4 populations with economic activities experienced partial or total lockdown lift. Scenarios L6 to L10 were defined similarly to L1 to L5 with all schools open and partial unlocking of unemployed and seniors. L11 to L15 represent the same situation as L6 to L10 with containment of contacts within categories (*wc*) or partial (half) containment of contacts within categories (*hwc*). This means that lockdown lift is adjusted to allow activities for specific days of the week depending on the subpopulation, leading to strictly limited inter-sub-population contacts. A mixed strategy was adopted with half within category contacts, limiting half of the contact between subpopulations thanks to a population-week regulation system (precise rules defining the combinations of exit authorizations depending on socio-professional category). In addition to the monitored scenarios L0-L15, a total lockdown exit at the start of phase 2 (scenario L99) was considered.

To better match the observed measures in the field, the monitored lockdown lift of phase 2 was combined with various options. The lockdown lift was implemented abruptly on May 11th (O1) or progressively at 4 or 8 weeks (O2 and O3). Because scenarios L1 to L15 cannot be applied indefinitely due to their economic and societal impacts, a third phase was created, and 2 other options were defined (based on O3 rules) to capture the long-term dynamics. Option O34 planned a total lockdown 2 weeks after the end of hospital saturation or after the peak of hospitalization if no saturation occurred. The total lockdown exit was definitive for O34 and was transitory for O35 (mixed strategy of lockdown lift and re-lockdown). The starting date of phase 3 consequently depends on the lockdown lift scenario.

**Figure 2:**
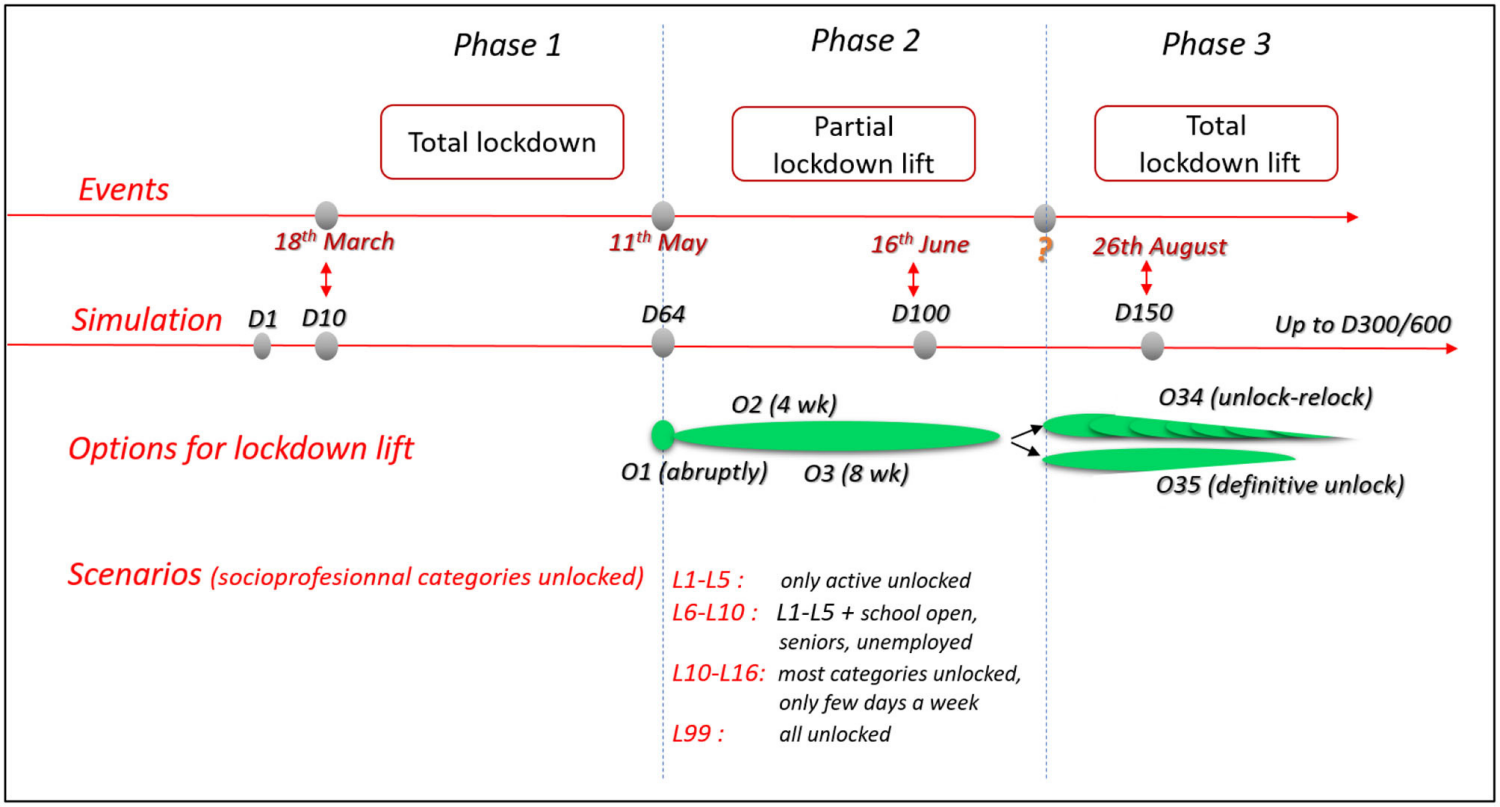
Flowchart of observed events and simulated options and scenarios for the 3 phases of the COVID-19 outbreak in the studied area.

Figure 2 summarizes the 3 phases of the French situation and the corresponding simulated lockdown exit strategies.

### Economic Optimization Model

Six economic scenarios (denoted E0 to E5) were considered (Table 2) for the 4 studied active populations locked down (Active_fixed, Active_lower, Active_intermediate, and Active_higher). During lockdown, the percentage of productivity compared to the pre-lockdown period is considered to vary depending on the socio-professional category (E0). This decrease in productivity is an average for the whole lockdown period (phase 1) and the subpopulation and should not be compared to productivity of workers with partial home working before lockdown. During the monitored lockdown lift (phase 2), the percentage of productivity compared to the pre-lockdown period was considered to depend on the percentage of activity released, in accordance with the lockdown lift scenario for a given subpopulation, as indicated in Equation (2):

(2) $$\begin{array}{l} {\text{Prod}}_{\text{Popi},\text{Ek}}\text{=}\{\begin{array}{c} \text{100 }*\;{\text{ActEco}}_{\text{Popi},\text{Ek }}\;*\;{\text{GDP}}_{\text{Popi},\text{Pl}},\;\\ if\text{ k=0 }\\ {}[{\text{ActEco}}_{\text{Popi},\text{E0}}\text{+}\left({\text{100-ActEco}}_{\text{Popi},\text{E0 }}\;\right)\\ {\text{ActEco}}_{\text{Popi},\text{Ek}};\text{ Max 100}]\;*\;{\text{GDP}}_{\text{Popi}},\;if\text{ k}>\text{0} \end{array} \end{array}$$

where:

Prod_Popi, Ek_ is the productivity permitted by the active population Pop *i* (Active_fixed, Active_lower, Active_intermediate, and Active_higher) for the economic scenario E*k* and the lockdown lift scenario L*l*
ActEco_Popi, Ek_ is the percentage of economic activity for the economic scenario E*k*
GDP_Popi_ is the daily GDP for the population Pop*i*.

**Table 2:** Economic scenarios and global production for the 4 active populations.

	**Active_lower**	**Active_fixed**	**Active_** **Intermediate**	**Active_higher**
**ECONOMIC SCENARIO ActEco**_**Popi, Ek**_				
E0	25%	0%	66%	66%
E1	ActRelease_Popi, Lj_			
E2	ActRelease_Popi, Lj_		ActRelease_Popi, Lj_ + 25%	ActRelease_Popi, Lj_
E3	ActRelease_Popi, Lj_			ActRelease_Popi, Lj_ + 25%
E4	ActRelease_Popi, Lj_ + 15%			
E5	ActRelease_Popi, Lj_ – 5%			
**INDIVIDUAL GLOBAL PRODUCTION GDP**_**Popi**_ **(**€ **PER DAY)**				
	326	326	423	571

Equation (2) aims to reproduce the fact that partial lockdown may help to improve economic activity compared to strict lockdown and that very good performance can be achieved with partial lockdown for some socio-professional categories.

Optimization under constraint was performed on the minimal total cost for Cost E_k_, L_j_, and hospitalization for the whole 300 or 600 d period. Economic risk was not accounted for. To combine the main key dimensions within the decision-making, the optimal solution that minimizes the overall economic impact for a given Tr was plotted considering 3 main constraints. Three levels of constraint were considered based on the quartile and median mortality rate observed between all the scenarios for a given option and a given Tr. The mortality criteria high, medium and low used for optimization correspond to no constraints on mortality, within the best half of the situation (lowest half mortality rate) and within the best quarter (lowest quartile mortality rate), respectively. The same type of rule was applied for the welfare criteria. The welfare criteria high, medium and low used for optimization correspond to within the best half or 75% of the number of person-days unlocked or no constraints on person-days unlocked, respectively. The criteria related to hospital saturation were defined by the duration of hospital saturation not to exceed or to the number of day-beds lacking for the whole period, with the criteria high meaning no constraints. The calculation of the total cost for each scenario and option allowed us to calculate the opportunity cost of choosing any combination of scenario and option compared to the scenario and option with the minimal cost for the whole period and given Tr.

### Model Parameterization

The number of contacts per person was defined within and between the 8 subpopulations, meaning *de facto* that contacts within and between the 3 epidemiologic populations (young, adults, and seniors) were considered. Hospitalization and admission to the ICU for severe cases were identified from Toulouse hospital data (29) and adjusted for the analyzed population. Hospitalization and ICU bed occupations were used to evaluate the capacity to welcome patients requiring these levels of care. The calibration of the compartmental model (Table 3) was performed similarly to Di Domenico et al. (25). At the beginning of the lockdown, other French areas were close to the hospital saturation level, and communication by the media raised peoples' awareness of health risks. We consequently consider that people changed their behavior dramatically for both the number of contacts during lockdown and Tr. As a consequence, the number of contacts within and between the subpopulations (Table 4) was based on previous publications (25, 30) and adjusted for the number of hospitalized and ICU patients during lockdown for the considered area. The simulated incidence of clinical cases was compared with the observed local incidence to appropriately adjust the number of contacts (Table 4). The value of Tr was likely to change with time during the studied period due to changes in rules, behaviors and protections availability, including masks. It was kept constant for a given simulation, and the values of 0.06, 0.10, 0.125, 0.20, and 0.25 were retained.

**Table 3:** Parameters, values, and sources to define the bioeconomic model.

	**Young**	**Students**	**Un-employed**	**Senior**	**Medical**	**Essentials**	**Active_** **lower**	**Active_** **fixed**	**Active_** **Intermediate**	**Active_** **higher**
Young	12.00	1.44	3.12	1.20	1.08	1.08	3.60	3.12	2.40	1.20
Students	4.32	3.36	2.64	1.68	0.45	0.69	2.04	2.64	2.64	2.52
Unemployed	4.32	3.36	2.64	1.68	0.66	0.45	2.04	2.64	2.64	2.64
Senior	0.30	0.60	3.12	8.40	0.42	0.42	2.40	2.40	1.68	0.12
Medical	4.32	3.36	2.64	1.68	0.66	0.45	2.04	2.64	2.64	2.64
Essentials	4.32	3.36	2.64	1.68	0.45	0.69	2.04	2.64	2.64	2.52
Active_lower	3.60	2.40	3.60	2.40	0.51	0.51	2.88	2.52	2.52	2.40
Active_fixed	3.12	2.4	4.44	2.40	0.66	0.66	2.52	4.56	1.20	0.48
Active_Intermediate	2.40	2.4	0.84	1.68	0.66	0.66	2.52	1.20	6.00	4.08
Active_higher	1.20	4.44	0.12	0.12	0.66	0.63	2.40	0.48	4.08	8.40

**Table 4:** Matrix contact (${\text{value of Contact}}_{\text{Popi}*\text{Pop}i^{\prime },Init}$) for the different populations.

**Variable**	**Description**	**Value**	**Source**
Θ^−1^	Incubation period	5.2 d	1
$\mu_{\text{p}}^{-1}$	Duration of prodromal phase	1.5 d, computed as the fraction of pre-symptomatic transmission events out of pre-symptomatic plus symptomatic transmission events	2
ϵ^−1^	Latency period	Θ^−1^ - $\mu_{\text{p}}^{-1}$	–
p_a_	Probability of being asymptomatic	0.2, 05	3
p_ps_	If symptomatic, probability of being paucisymptomatic	1 for children 0.2 for adults, seniors	4
p_ms_	If symptomatic, probability of developing mild symptoms	0 for children 0.7 for adults 0.6 for seniors	4
p_ss_	If symptomatic, probability of developing severe symptoms	0 for children 0.1 for adults 0.2 for seniors	4–6
s	Serial interval	7.5 d	7
μ^−1^	Infectious period for I_a_, I_ps_, I_ms_, I_ss_	S - Θ^−1^	–
r_β_	Relative infectiousness of I_p_, I_a_, I_ps_	0.51	8
p _ICU_	If severe symptoms, probability of going in ICU	0 for children 0.36 for adults 0.2 for seniors	9
λ _H, R_	If hospitalized, daily rate entering in R	0 for children 0.072 for adults 0.022 for seniors	9
λ _H, D_	If hospitalized, daily rate in D	0 for children 0.0042 for adults 0.014 for seniors	9
λ _ICU, R_	If in ICU, daily rate entering in R	0 for children 0.05 for adults 0.036 for seniors	9
λ _ICU, D_	If in ICU, daily rate entering in D	0 for children 0.0074 for adults 0.029 for seniors	9

The assumptions on social distancing intervention made by (25) were kept. A 75% decrease in the number of contacts is expected if severe symptoms are observed in one individual. Five percent of adults stayed at home in the case of school closures, with the exception of the medical and essential activities subpopulations. Working from home was adopted by 6% of the active adult population before the lockdown. The isolation of positive cases when returning home was not considered as possible for phase 1, in accordance with the main observations during this phase. The number of beds available for hospitalization and ICU was 1,000 and 300, respectively (29). A higher number of patients hospitalized or in the ICU on a given day defined the saturation situation, which was associated with a three-fold higher mortality risk for people above the threshold. The price per day-bed was fixed to 500 € and 1,500 € for hospitalization and ICU, respectively (31).

The parameters of the six economic scenarios are reported in Table 2. The range of activity during lockdown compared to the pre-lockdown period was considered to vary between 0 (fixed) and 66%. This means, for instance, that the productivity of a home worker is 66% of his or her former productivity. A sensitivity analysis is permitted with scenarios E2 to E4, which attribute a fixed extra percentage of productivity, and in scenario E5 (limited productivity even if there is a high rate of lockdown lift).

Daily GDP was obtained as the yearly GDP per worker [€77,212 in 2018 for the Occitanie area (32)] and adjusted for each subpopulation due to variation in the official estimation of socioprofessional standard living incomes (27). The local standard living incomes were officially assessed as €18,870, €18,870, €24,520, and €33,090 for the socio-professional categories Active_low, Active_fixed, Active_intermediate, and Active_high, respectively. The yearly GDP per worker for each socio-professional category was then divided by 200 days worked yearly (Table 2).

We calibrated our model with demographic and socioeconomic data describing Toulouse area i.e., a French metropoly with a relatively high level of economic activity and several universities and higher education structures. Our findings may not be extrapolated to other cities, as the parameters may vary between cities. However, many of the cities with similar sizes in Europe would likely have close levels of healthcare and university facilities. To some extent, our results provide valuable information for scientists and policy-makers beyond Toulouse area. At least we laid down in this empirical application the rationale and the elements required to implement a tailored and adaptive approach of COVID-19 management.

## Results

The results are presented for a 300- and 600-day period simulation to represent the short- and mid-term impacts. The lockdown starts on day 10 of the simulation (18th March 2020), and the lockdown lift starts on day 64 (11th May 2020). Day 100 corresponds to mid-June 2020, and day 150 corresponds to late August 2020 (Figure 2).

### Validation and Sensitivity of the Bioeconomic Model

**Figure 3:**
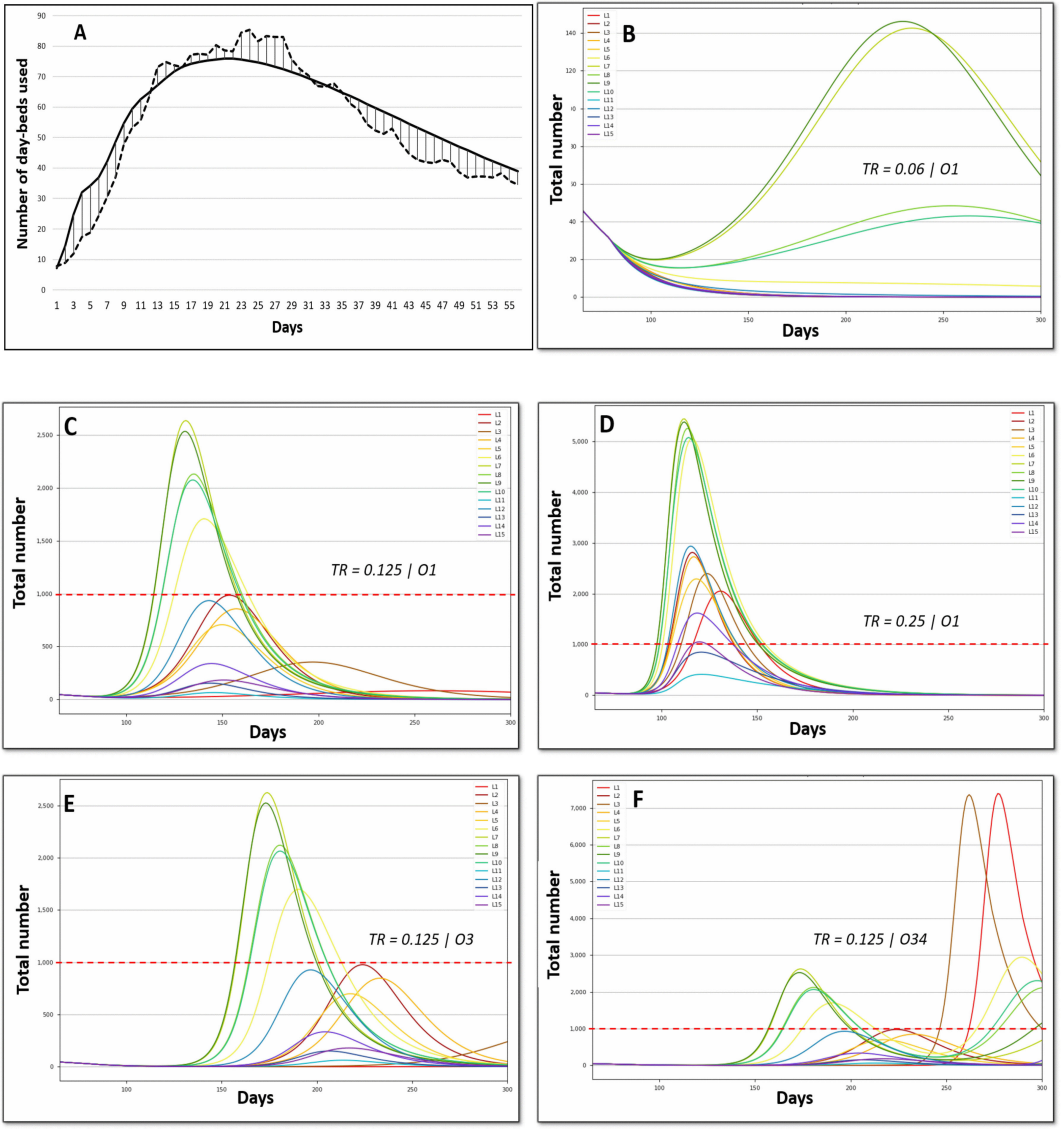
Epidemiologic validation of the model. **(A)** Comparison between the predicted (solid line) and observed (dashed line) number of day-beds used. **(B–F)** Number of daily beds used in hospital (CIU excluded). The red dashed line represents the hospital capacity. Tr, transmission rate; Option 1, abrupt monitored lockdown lift early in phase 2; Option 3, progressive monitored lockdown lift on 8 weeks; Option O34, progressive total lockdown lift (phase 3).

The validation of the epidemiological part of the model was based on the comparison between the simulated and observed number of day-beds used, with a high match observed (Figure 3A). The results were highly sensitive to Tr, as illustrated for option O1 in Figures 3B–D: the number of day-beds used was very low for a Tr of 0.06 and increased dramatically when Tr increased to 0.125 and 0.25. Because the change in Tr represents the average population behavior around virus transmission in our model, the results are presented for these 3 values of Tr. The results also highlight the capability of the scenarios to represent various situations in terms of outbreak dynamics for the different phases (Figures 3B–D). For instance, hospital saturation may be prevented by some combination of Tr and scenarios, whereas other combinations lead to long and intense hospital saturation.

For a given Tr (Figures 3C,E for Tr = 0.125), extending the lockdown lift by 8 weeks, as occurred in France for most activities, postponed the peak (to a greater degree when the peak was low) but failed to reduce the peak intensity. The adoption of a total lockdown leads to a second wave. For a lockdown simulated at 8 weeks and Tr = 0.125 (Figure 3F, O34), the second wave starts on day 250. This clearly shows the need for long-term consideration to improve multi-criteria decisions.

**Figure 4:**
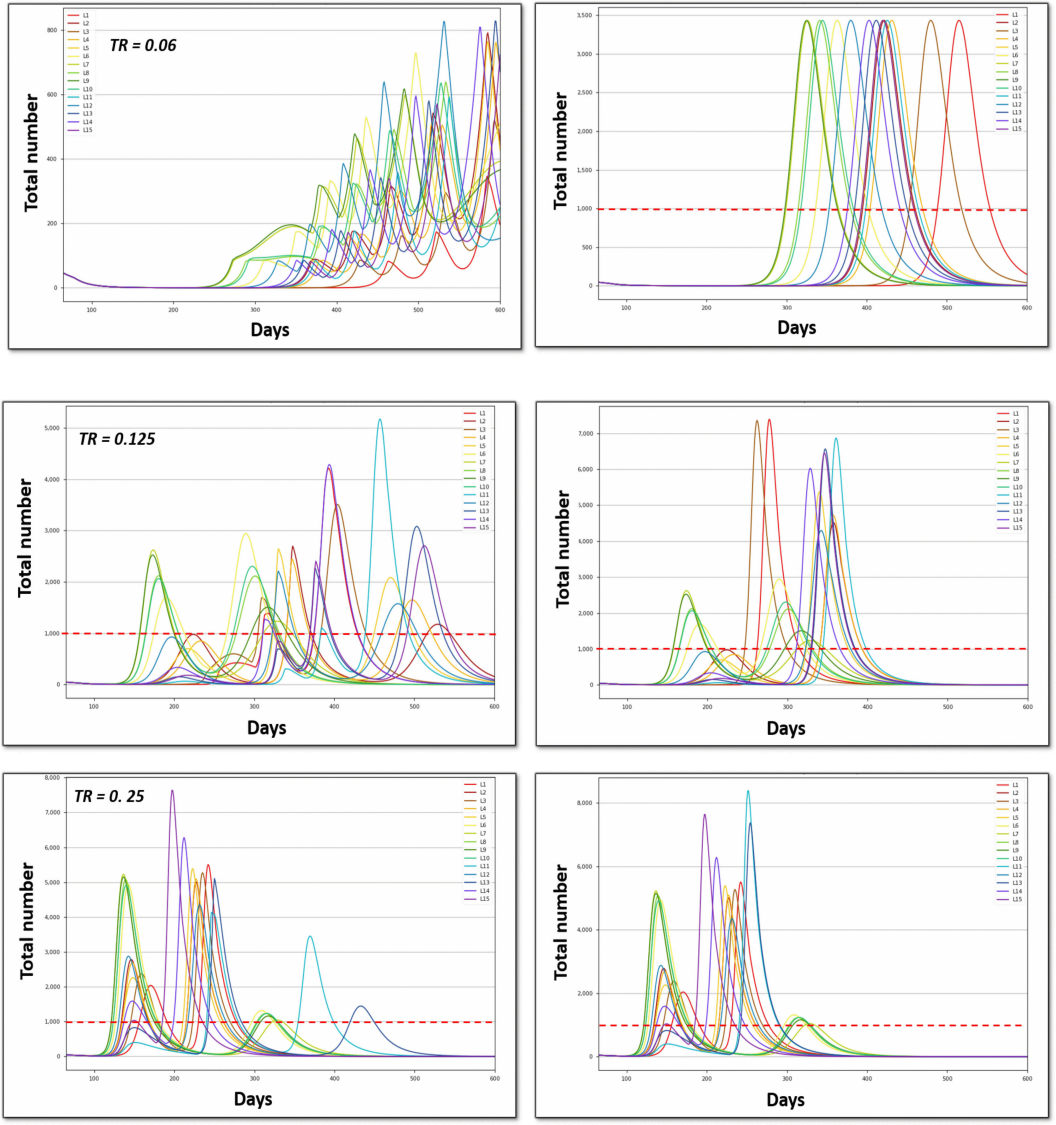
Number of daily beds used in the hospital (ICU excluded) for the different scenarios (L1 to L15). Tr, transmission rate; the red dashed line represents the hospital capacity. Option O34 (left), progressive total lockdown lift (phase 3); Option O34 (right), abrupt total lockdown lift (phase 3).

Options O34 and O35 were consequently modeled up to 600 days (Figure 4). On the one hand, the greater the Tr (top to down for a given column), the earlier and the higher the peak for both the strategy of lockdown lift and re-lockdown (O34, left) or the total definitive lockdown strategy (O35, right). Hospital saturation was only avoided when Tr remained very low (Tr = 0.06) and with lockdown lift and re-lockdown strategies (O34). These results demonstrate that individual behavior (i.e., the Tr value) is more important than political strategy (scenario choices) for the long-term overall impact. On the other hand, for a given Tr (i.e., given an average behavior), scenario choices permitted the management of the hospital saturation level, including intensity and duration.

### Multidimensional Long-Term Optimization

**Figure 5:**
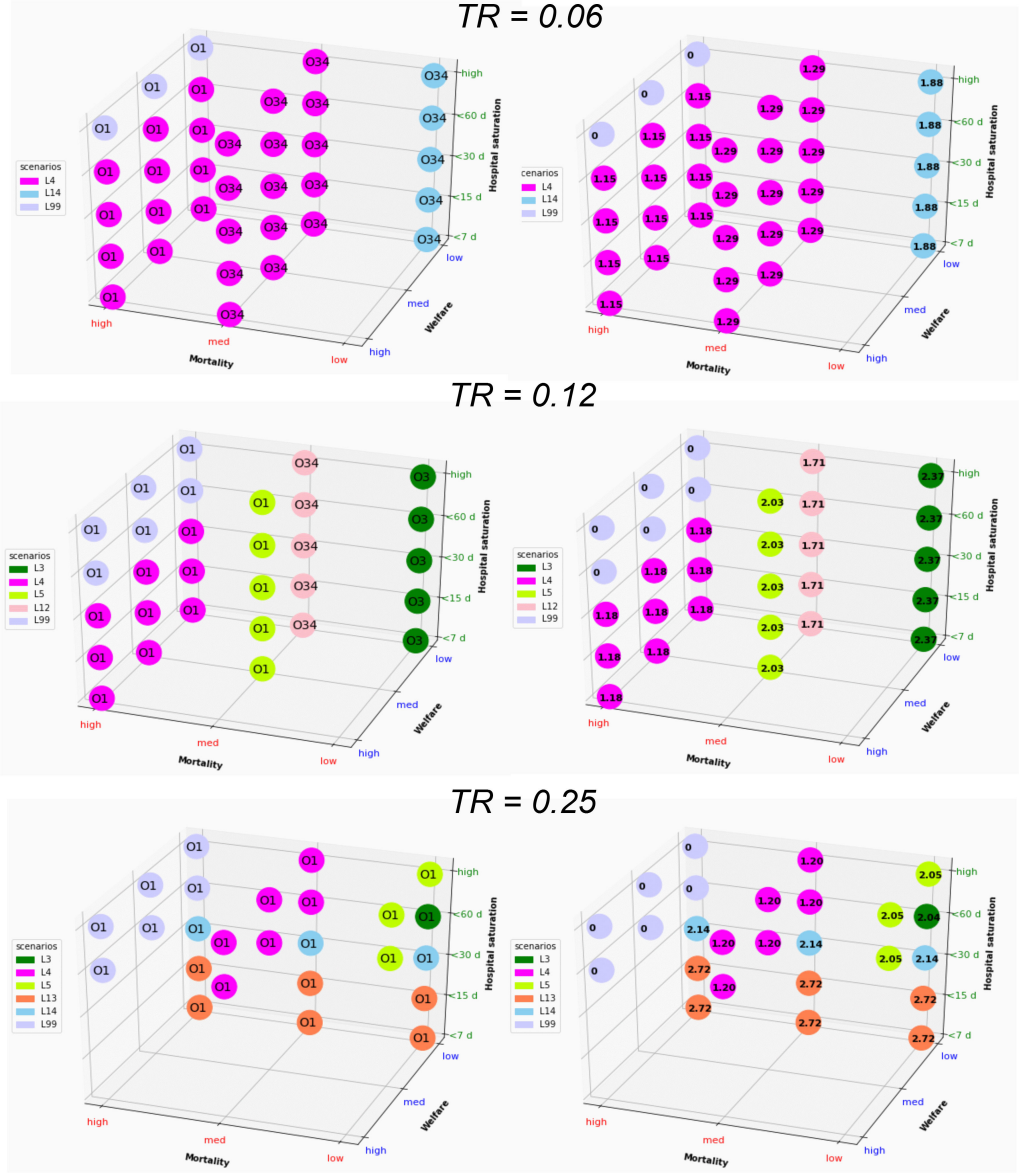
Graphical representation of the optimal solution depending on the strength of the constraint for a whole period of 300 days. The results in the right column are expressed as direct cost (in billion euros); Tr, transmission rate. The optimal solution that minimizes the overall economic impact under a set of constraints is found in the foreground (low mortality, high welfare, and low saturation).

**Figure 6:**
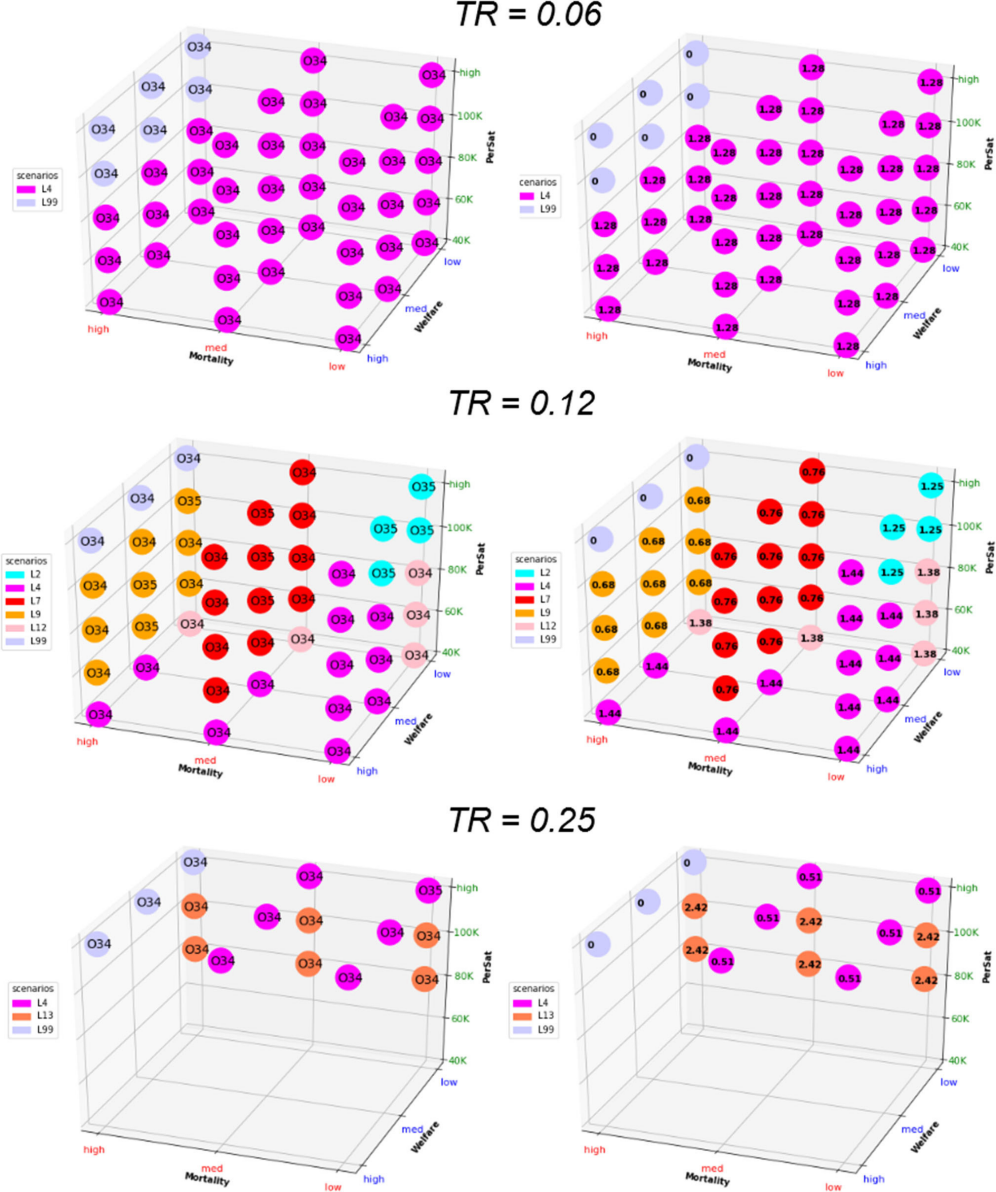
Graphical representation of the optimal solution depending on the strength of the constraint for a whole period of 600 days. The results in the right column are expressed as direct cost (in billion euros); Tr, transmission rate. The optimal solution that minimizes the overall economic impact under a set of constraints is found in the foreground (low mortality, high welfare, and low saturation).

The optimal solutions under different levels of constraints are reported in Figures 5, 6, Supplementary Figures 1, 2. The solution that minimizes the overall economic impact is located in the foreground of Figures 5, 6, Supplementary Figures 1, 2 (high welfare, low mortality and low saturation). The results indicate the name of the scenario and option as well as the corresponding direct total cost compared to the reference (opportunity cost value = 0). The total cost of lockdown strategies for O34 and the 300-day period was €2.15 billion for L99 and varied from €3 to €6 billion for scenarios L1-L15.

Short-term optimization (Figure 5) shows that improving one criterion necessarily leads to deterioration of another criterion. Combining L4 or L14 and O3 represents the best strategy in most of the situation for low Tr, with an opportunity cost of €1.29–€1.88 billion for the 300 d period. For higher Tr, options O1, O3, and O34 and scenarios L3, L4, L5, L12, L13, and L14 appear to be optimal providing that constraints are released on at least 2 criteria. The direct cost of optimal scenarios to improve welfare or to decrease mortality and hospital saturation vary between €1.2 and €2.7 billion (L99 excluded).

Considering the long-term impact (Figure 6) leads to dramatically different results, with Option O34 as the best solution except for very few situations. The lockdown lift and re-lockdown strategies fulfill the 3 constraint criteria for at least Tr = 0.06 and 0.125. For Tr = 0.25, neither welfare nor saturation constraints may be completed. The best scenarios for long-term optimization are L4 for Tr=0.06 (similar to short-term analysis), L4 and L13 for TR = 0.25 and L2, L4, L7, L9, and L12 for TR = 0.125. The opportunity cost is €1.28 billion for Tr = 0.06, €0.68–€1.44 billion for Tr = 0.125 and €0.51–€2.42 billion for Tr = 0.25. Very similar results are observed when formulating the saturation constraints in terms of saturation duration without the condition of saturation intensity (Supplementary Figure 1) instead of per patient-saturated numbers (Figure 6).

The second or third best optimal strategy (Supplementary Figure 2) is consistently found to be option O34 combined with scenarios L2, L3, L4, L5, L7, L8, L9, and L12. The opportunity cost compared to the first best solutions for each set of constraints is small to very small, showing little difference linked to the choice of the scenario within this range of scenarios.

## Discussion

The present work is the first long-term bioeconomic multicriteria optimization approach applied to COVID-19 at a local scale and was conceived to support decision-making regarding public health policy. Various criteria were considered within the economic part of the model, and the epidemiologic complexity of the situation was simplified. The present approach allows us to consider 8 socio-professional categories and 3 epidemiologic populations at a glance.

Unlike other studies, the present work focuses on a population with very limited virus circulation before lockdown. The situation in Paris and Eastern France in February and March 2020 as well as the situations in other places in Europe or worldwide clearly highlight the medical consequences of the virus, spontaneously leading to a moderate to high level of barrier routines in daily activities for both professional and non-professionals. The contact matrices and the contamination probability were adjusted accordingly in the present simulation. Because lockdown froze all professional and private activities of the majority of the population, the simplified version of the SIR modeling provided here was precise enough to predict the number of cases observed in hospitals. The value of Tr to be retained within the bioeconomic model is a key point, and all the results demonstrate that identification of the best solution is highly sensitive to this parameter. It represents individual behaviors related to barrier gestures and individuals' compliance with biosecurity and social distancing rules. Its true value is consequently very difficult to appraise and may even change between socio-professional categories (e.g., education, information asymmetry). The fixed Tr value for a given set of scenarios and options is an important simplification of the present study since Tr is likely to change over time. The options offered to people to protect themselves (disinfectant gel, masks, etc.) and the rules or recommendations provided by authorities may influence the value of Tr. For instance, in France, masks were available for everyone at the beginning of phase 2, and the recommendation of mask wearing has changed over time (first on a voluntary basis and in mid-summer, mandatory in all closed public rooms and outside in some crowded tourist towns). Moreover, the sensitivity of people was limited when prevalence was low in France and Europe but then increased during summer 2020, when the second wave was observed in other European countries and movement bans reappeared.

The progressive lockdown lift, as simulated for 8 weeks in option O3, is very close to the field situation. When phase 2 started, the practical application of the lockdown lift took 1–2 months as many offices, schools and day cares were not in a situation to host people. High schools and universities remained closed up to September 2020. Most social activities (museums, restaurants, pubs and cinemas) opened progressively from May to August 2020. Traveling was first authorized within 100 km of home, and then free circulation was authorized. The ability of the present model to closely represent the lockdown lift strategy can be considered high. A limitation of our model is that it does not account for summer breaks and the higher rate of movement and contact that may take place during this specific period.

The present work used a simplified vision of economic dynamics by summarizing the creation of value by each actor to his or her daily contribution to the GDP before lockdown. A global and dynamic approach of the industrial and economic activities that could be permitted by economic global (or partial) equilibrium modeling or the equivalent may provide a precise approach to guide decision making. It may help to better consider the interrelationship between sectors and the dependency between actors and post-disaster recovery dynamics. It is difficult to truly appreciate the relationship between the sectors and all the information required to parametrize the models during the recovery phase due to the paucity of updated information. This means that most of the calibration would be based on an assumption of business as usual. Using GDP per socio-professional category is a strong assumption, and GDP is clearly a very raw proxy of the state of the economy. However, it allows the combination of SIR outbreak modeling and economic societal considerations within a unique decision-making process, which clearly adds value compared to previous studies.

The main results of the present work are that policy makers should focus more on individual behaviors (represented by the Tr value) than on trying to optimize lockdown strategy (defining who is unlocked and who is locked). Social distancing is recognized as a key parameter to limit the spread of diseases but is often associated with high economic impact (17). The main challenge is therefore to maintain social distancing by appropriate individual behaviors without excessive coercive government-enforced social distancing, which is very often associated with high economic impact. In countries with poor socioeconomic conditions, stringent social distancing measures and generous income support programmes have been shown to lower cases and deaths (33). These findings suggest that evaluating the global impact of COVID-19 or optimization to define the best strategy may represent a priority and that research in compartmental economics or experimental economics may be needed to address COVID-19 issues. A better understanding of individual behavioral motivations and the identification of ways to improve biosecurity compliance for everyone should become the short-term priority.

The results clearly show that no major differences in the economic impact or in the 3 criteria retained can be seen between the scenarios. Scenarios L4 and L13 appear to be the best, and scenarios L2, L3, L5, L7, L14, and L12 can also be considered as multi-criteria equivalents. The L1, L6 L10, L11, L15, or L15 scenarios should not be recommended. The scenarios that limit interactions between socio-professional categories, which can be seen as precision lockdown lift scenarios (L11 to L15), were expected to represent the best trade-off between the constraints, but they failed to ensure satisfactory welfare criteria, with the overall outdoor access limited compared to other scenarios.

In all the potentially recommended scenarios, the hospital saturation level was handled with regard to both intensity and duration. Although we demonstrate here that several criteria may be considered simultaneously for decision-making and that hospital saturation and the associated mortality increased risk cannot justify an endless strict lockdown, public health remains the most important criterion in the short term, and the scenarios contribute to its optimization. Hospital saturation is not only a public health issue but also a key political risk of lockdown policy rejection (2).

In conclusion, our results demonstrate that policy makers should focus on individuals' behavioral changes rather than on trying to optimize lockdown strategies (defining who is unlocked and who is locked). The results highlight the need for compartmental or experimental economics to address COVID-19 issues through a better understanding of individual behavioral motivations and the identification of ways to improve biosecurity compliance.

## Data Availability Statement

Publicly available datasets were analyzed in this study. This data can be found at: https://www.data.gouv.fr/fr/datasets/?q=covid&page=1.

## Author Contributions

AF, GL, and DR designed the study. AF and DR performed the modeling (model calibration, and Python and Gams code). AF and RB performed the simulations. AF, RB, GL, and DR analyzed the results. GL and DR wrote the manuscript. All authors contributed to the article and approved the submitted version.

## Conflict of Interest

The authors declare that the research was conducted in the absence of any commercial or financial relationships that could be construed as a potential conflict of interest.
