# Supplementary material for: Individual Behaviors and COVID-19 Lockdown Exit Strategy: A Mid-Term Multidimensional Bio-economic Modeling Approach
Source: Front Public Health. 2020 Nov 17;8:606371. doi: 10.3389/fpubh.2020.606371 (PMC7705347; doi:10.3389/fpubh.2020.606371)

**Supplementary Figure 1 : Graphical representation of the optimal solution depending on the strength of the constraint for a whole period of 600 days, with saturation expressed in duration (days above hospital capacity).** The results in the right column are expressed as direct cost (in billion euros); Tr: transmission rate. The optimal solution that minimizes the overall economic impact under a set of constraints is found in the foreground (low mortality, high welfare and low saturation).


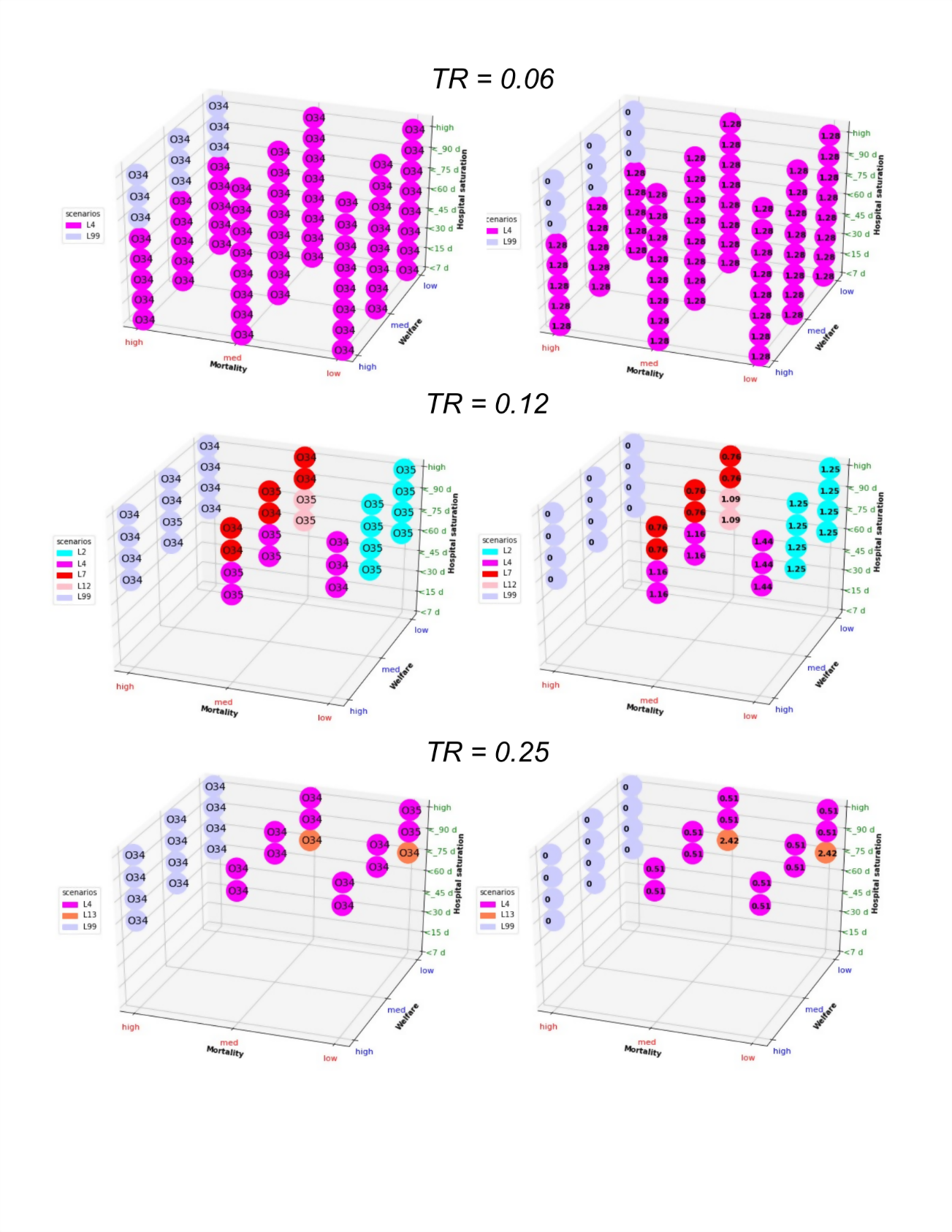


**Supplementary Figure 2 : Graphical representation of the second (left) and third (right) optimal solutions depending on the strength of the constraint for a whole period of 600 days.** The results are expressed in opportunity cost compared to best for second best or compared to second best for third best solutions; Tr: transmission rate. The optimal solution that minimizes the overall economic impact under a set of constraints is found in the foreground (low mortality, high welfare and low saturation).


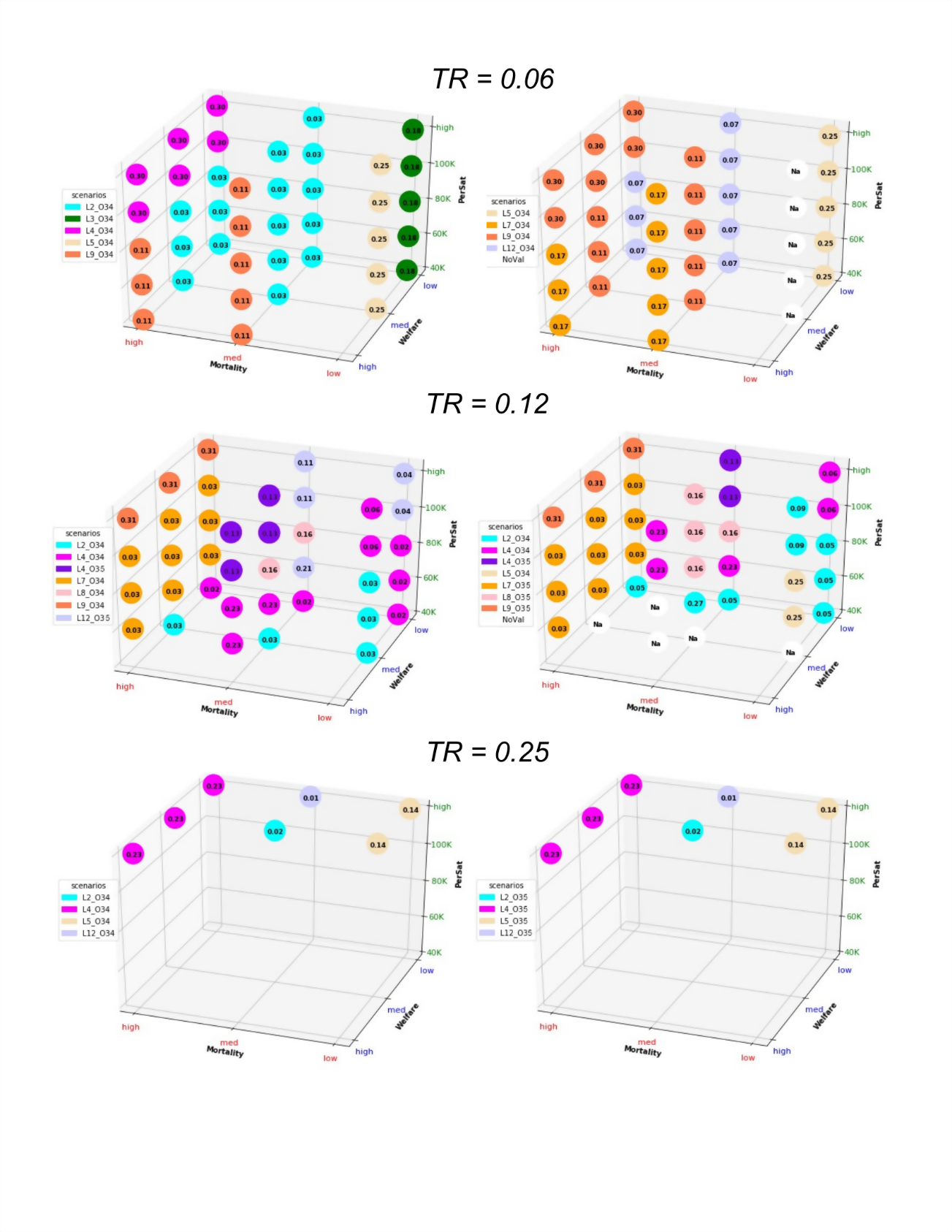

Supplement: Supplementary file 1 [file Data_Sheet_1.docx]
